# Assessment of Retinoblastoma Capacity in the Middle East, North Africa, and West Asia Region

**DOI:** 10.1200/GO.20.00321

**Published:** 2020-10-16

**Authors:** Michala Burges, Ibrahim Qaddoumi, Rachel C. Brennan, Lisa Krull, Natasha Sahr, Carlos Rodriguez-Galindo, Sima Jeha, Matthew W. Wilson

**Affiliations:** ^1^ Department of Global Pediatric Medicine, St Jude Children’s Research Hospital, Memphis, TN; ^2^ Hamilton Eye Institute, Department of Ophthalmology, University of Tennessee, Memphis, TN; ^3^ Department of Oncology, St Jude Children’s Research Hospital, Memphis, TN; ^4^ Department of Statistics, St Jude Children’s Research Hospital, Memphis, TN; ^5^ Department of Ophthalmology, St Jude Children’s Research Hospital, Memphis, TN

## Abstract

**PURPOSE:**

We aimed to evaluate the capacity to treat retinoblastoma in the Middle East, North Africa, and West Asia region.

**METHODS:**

A Web-based assessment that investigated retinoblastoma-related pediatric oncology and ophthalmology infrastructure and associated capacity at member institutions of the Pediatric Oncology East and Mediterranean group was distributed. Data were analyzed in terms of availability, location, and confidence of use for each resource needed for the management of retinoblastoma. Resources were categorized by diagnostics, focal therapy, chemotherapy, advanced treatment, and supportive care. Responding institutions were further divided into an asset-based tiered system.

**RESULTS:**

In total, responses from 23 institutions were obtained. Fifteen institutions reported the availability of an ophthalmologist, 12 of which held primary off-site appointments. All institutions reported the availability of a pediatric oncologist and systemic chemotherapy A significant portion of available resources was located off site. Green laser was available on site at seven institutions, diode laser at six institutions, cryotherapy at 12 institutions, and brachytherapy at nine institutions. There existed marked disparity between the availability of some specific ophthalmic resources and oncologic resources.

**CONCLUSION:**

The assessment revealed common themes related to the treatment of retinoblastoma in low- and- middle-income countries, including decentralization of care, limited resources, and lack of multidisciplinary care. Resource disparities warrant targeted intervention in the Middle East, North Africa, and West Asia region to advance the management of retinoblastoma in the region.

## INTRODUCTION

Retinoblastoma is a rare intraocular malignancy; however, > 8,000 children are diagnosed every year worldwide with this neoplasm.^1^ In developed and high-middle–income countries, early recognition of disease, highly specific ophthalmic and oncologic resources, and strong multidisciplinary teams have led to > 90% survival.^1-3^ However, in low- and middle-income countries (LMICs), late diagnosis, limited accessibility, lack of treatment-specific resources, and poor compliance result in survival rates < 40%.^2,4^ More than 80% of the estimated 160,000 children with pediatric cancer reside in developing countries; therefore, more children die of retinoblastoma than survive globally.^5,6^ The disproportionate burden of disease motivates the need to improve retinoblastoma survival in LMICs.

**CONTEXT**

**Key Objective**
What is the retinoblastoma-specific capacity of institutions belonging to the Middle East, North Africa, and West Asia region in terms of ophthalmic and oncologic resources?
**Knowledge Generated**
The assessment revealed disparity between ophthalmic and general oncology resources, allowing us to separate respondent institutions into an asset-based classification system. The majority of institutions belonged to the middle tier (tier 2), which requires the capacity to treat disease by enucleation with limited option for eye-sparing therapy as a result of lack of focal modalities.
**Relevance**
To our knowledge, this is the first assessment of its kind, one dedicated to the capacity to treat a rare pediatric cancer that requires highly specialized resources. With knowledge of resource capacity and subsequent institution classification, an informed referral network and strategy can be designed for the region. In addition, this methodology can serve as a model for survey examination of other rare pediatric cancers that similarly require highly specialized resources.

Multidisciplinary care is integral to successful management of retinoblastoma. Pediatric oncologists, pathologists, and radiation oncologists, as well as basic diagnostics and a therapeutic infrastructure, are inherent cancer center components. However, ophthalmology is a restricted global resource with limited availability, further reduced when subspecialty training (ocular oncology) is considered.^4^ Ophthalmic infrastructure, including cryotherapy, laser therapy, fundus camera, and options for locally delivered chemotherapy, is linked with high equipment costs. Thus, the treatment of retinoblastoma necessitates the acquisition of both personnel and equipment that extend beyond basic pediatric cancer care.

The Pediatric Oncology East and Mediterranean (POEM) group is a collaborative platform established in 2013 for health care professionals from pediatric oncology centers in 28 countries in the Middle East, North Africa, and West Asia region. The goal of the group is to share experience and establish common strategies to optimize the care of pediatric oncology patients. Working with POEM, we conducted the first assessment of relevant retinoblastoma-related treatment capacity in the Eastern Mediterranean region. This comprehensive needs assessment is a crucial first step in improving global survival of retinoblastoma by developing targeted, regional strategies for disease management.

## METHODS

A Web-based assessment (SurveyMonkey, San Mateo, CA) that investigated retinoblastoma-related pediatric oncology and ophthalmology infrastructure was developed with expert review by an ocular oncologist and three pediatric oncologists with experience in the management of patients with retinoblastoma. Concern for self-reported outcomes bias was identified, and the survey was refined to focus explicitly on resource infrastructure.

A Web link^7^ was distributed to representatives from POEM member institutions (n = 72). There were 15 questions in total, eight of which had multiple components. Availability, on-site/off-site location, and confidence of use were examined for 30 resources via the eight multiple-component questions. Three reminder e-mails were sent requesting the completion of the assessment from June to September 2018. Respondents were required to answer every question from a drop-down menu before submission to mitigate response bias.

Resources were grouped into the following categories: human resources, diagnostics, and treatment modalities. A range from general oncology to highly specific retinoblastoma treatment modalities was delineated (Tables 1 and 2) to evaluate centralization of care, training opportunities, and the availability of resources across the region.

**TABLE 1:**
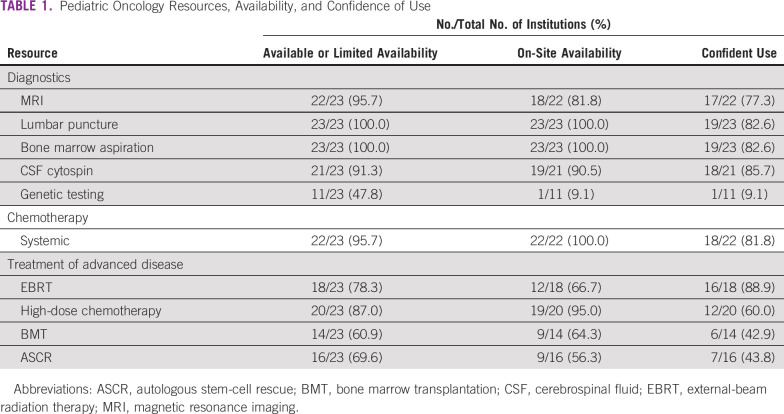
Pediatric Oncology Resources, Availability, and Confidence of Use

**TABLE 2:**
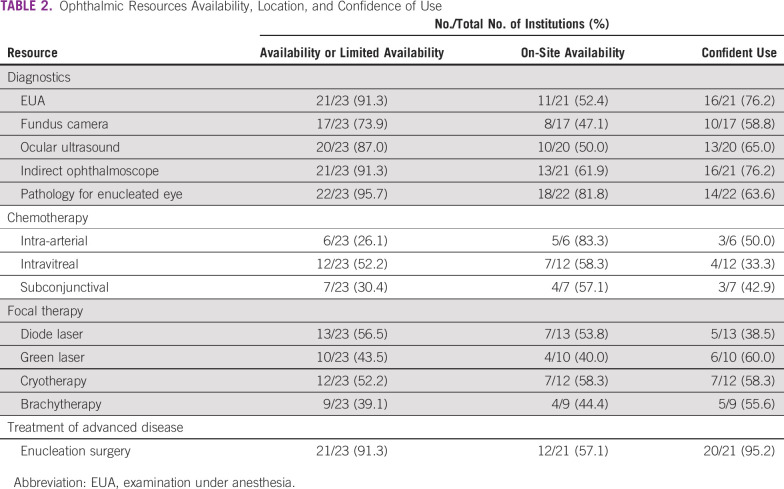
Ophthalmic Resources Availability, Location, and Confidence of Use

Respondent institutions were categorized into an asset-based tier classification system based on availability of ophthalmic recourses that was aided in part by the International Society of Pediatric Oncology–Pediatric Oncology in Developing Countries guidelines for retinoblastoma.^6^ Tier 1 did not have access to an ophthalmologist to treat retinoblastoma. Tier 2 had access to an ophthalmologist. Tier 2 had access to enucleation, chemotherapy, and ophthalmic pathology, but a lack of focal treatment modalities limited opportunities for ocular salvage. Tier 3 had access to enucleation and eye-sparing therapies for treatment of intraocular disease, including access to most focal therapies.

Descriptive statistics (frequency and percentage) were reported for survey questions. The Cochran-Armitage trend test was used to assess the association between institutional ophthalmic and basic oncologic resources (no or yes and available or not available) and institutional tier classification (tiers 1, 2, and 3). Exact *P* values were reported for these associations. *P* values were adjusted for multiple testing but are not reported. We also explored the association between institutional ophthalmic and basic oncologic resources and institutional tier classification adjusting for gross domestic product (GDP) using analysis of covariance and logistic regression. These analyses revealed no impact of GDP and therefore provide no additional information. Therefore, we only report on the bivariate associations. Statistical analyses were conducted using SAS software version 9.4 (SAS Institute, Cary, NC). A two-sided significance level of *P* < .05 was considered statistically significant.

## RESULTS

**FIG 1:**
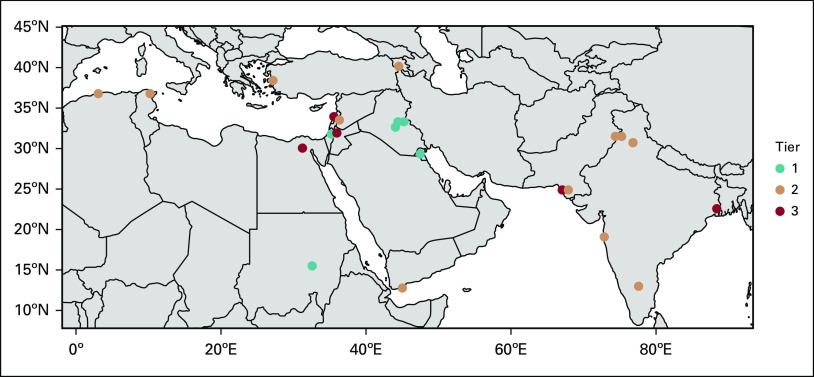
Geospatial representation of tiers.

The needs assessment survey was sent via Web link to 72 POEM-affiliated institutions, with 27 respondents. Four respondents were excluded (two submissions had no survey question responses, and two were duplicate responses from the same institution). In the duplicate cases, the most complete response was selected from each institution. Twenty-one of 23 analyzed responses were complete; two respondents submitted incomplete forms as a result of a system error. The 23 respondents represent 15 countries and 32.0% of the POEM member institutions at the time of distribution (Fig 1). According to the World Bank income level classification, one respondent belongs to a high-income country, eight respondents belong to upper-middle–income countries, 12 respondents belong to lower-middle–income countries, and two respondents belong to low-income countries.^8^

Key personnel were reported, including those with primary off-site appointments, which indicates the time and effort of the provider were divided between two or more institutions as a consulting service. Pediatric oncologists were present at all 23 institutions, five (21.7%) of which held primary off-site appointments. Pathologists were available at 20 (87.0%) institutions, six (30.0%) of which held primary off-site appointments. Radiation oncologists were present at 17 (73.9%) institutions, nine (52.9%) of which held primary off-site appointments. Ophthalmologists were present at 15 (65.2%) institutions, 12 (80.0%) of which held primary off-site appointments.

The association between ophthalmic or oncology resources and the tier classification system was analyzed. Statistically significant differences were found regarding availability of ophthalmologists, clinical nurse coordinators, ophthalmic supportive staff, occupational therapy, ophthalmic ultrasound, diode laser, cryotherapy, intravitreal chemotherapy, and subconjunctival chemotherapy (*P* < .001; Table 3). Other individual resources showed no statistically significant differences across tiers. Several oncology resources were robustly present across all tiers, such as pediatric oncologists, systemic chemotherapy, magnetic resonance imaging (MRI), lumbar puncture, bone marrow aspiration, and cytospin. Ophthalmologic resources with a high prevalence across all tiers were examination under anesthesia, fundus camera, indirect ophthalmoscopy, and pathology. However, resources such as a genetics, brachytherapy, and intra-arterial chemotherapy were proportionally similar across all tiers, but the overall availability of these resources was diminished, with only five, nine, and six centers reporting access to the resource, respectively.

**TABLE 3:**
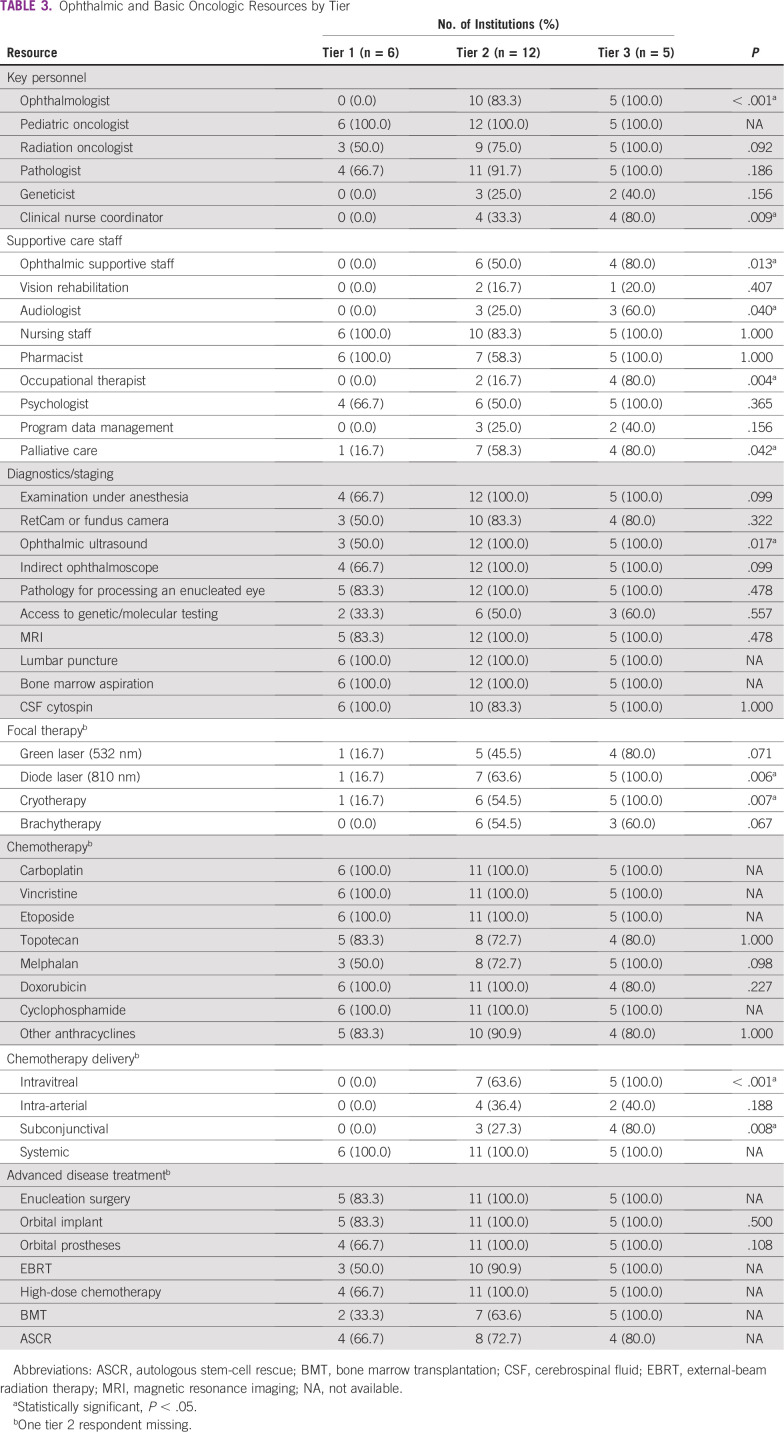
Ophthalmic and Basic Oncologic Resources by Tier

## DISCUSSION

A clear understanding of retinoblastoma-specific capacity has been lacking within the Eastern Mediterranean region. To our knowledge, this assessment represents the first comprehensive, resource-based survey dedicated to identifying capacity in a region for management of a specific pediatric cancer that requires multidisciplinary care. The novelty and specificity of responses provide informed recommendations with a degree of confidence for targeted intervention to improve the management of patients with retinoblastoma within the region.

Centralization of resources and cohesive multidisciplinary teams remain the primary gaps in patient care. All 23 POEM institutions had at least one pediatric oncologist; however, only 15 institutions had an ophthalmologist available. Of these 15 ophthalmologists, 12 had primary practices off site. This creates a disparity between oncologic and ophthalmologic care and indicates that patients with retinoblastoma are being treated at oncology units where ophthalmology may not be primarily available. The decentralized services place an additional travel burden on families and may impact rates of abandonment of therapy.

The partnership between pediatric oncology and ophthalmology is critical to the successful management of patients with retinoblastoma. The lack of ophthalmologists and ophthalmic infrastructure identified in this survey indicates a disparity between these subspecialties. This was noted by the comparison showing a median on-site availability of seven ophthalmology resources compared with 17.5 oncology resources (Tables 1 and 2). Although ophthalmic resources can have an aggregate cost of approximate US$250,000, consortium or in-bulk purchasing can mitigate overall costs and begin a network of collaboration in the region to overcome this disparity.

Building disease-specific expertise can overcome limited resources and centralize care. Elzomor et al^9^ showed improved patient outcomes after implementation of multidisciplinary care and protocol-based treatment in Egypt. Improved outcomes included a decrease in mean time between enucleation and diagnosis, an increase in length of optic nerve obtained surgically, and an increase in the probability of survival.^9^ There is abundant literature correlating improved outcomes to centralized services and increased patient volume,^10,11^ such as centralization of pediatric neuro-oncology care leading to greater tumor resections with fewer complications,^12^ lower ventricular shunt failures,^13^ and lower mortality after craniotomy.^14^

Centralization of retinoblastoma care could similarly improve surgical outcomes such as a longer length of optic nerve resected during enucleation^9^ and familiarization with indications and use of focal eye-sparing modalities, such as laser, cryotherapy, and plaque brachytherapy. Centralized hospitals have also proven to be more cost effective because pediatric oncology resources can be used to treat all cancers and needed ophthalmic infrastructure can be used to care for a greater number of patients.^11^ Al-Haddad et al^15^ increased patient volume from 20 patients between 2002 and 2011 to 52 patients between 2012 and 2017 by establishing a centralized service with increased referral from neighboring countries. In the case of our assessment, centers within the same city or country should identify one center to treat retinoblastoma and refer accordingly. Centralization, however, does come with a cost because it places a greater treatment burden on patients and families by increasing the distances traveled for care.^16^

Enucleation by a skilled ophthalmologist can cure most patients with localized, unilateral intraocular disease and should remain the standard of care when stringent follow-up is not guaranteed.^17^ In addition, excellent cosmesis is possible when orbital implants and protheses are available. On the basis of the assessed resource gaps, enucleation is the only available curative treatment in 13 centers as a result of lack of available of eye-sparing therapies.^18-20^ However, enucleation refusal is particularly common in LMICs, especially in contrast to high-income countries,^20^ as a result of social stigma, lack of support for visually impaired, belief in alternative medicine, religious or parental beliefs, and cultural contexts.^20^ Inability to offer complete cosmetic rehabilitation can further exacerbate enucleation refusal in the 30% of centers that reported limited or no access to orbital implants and 31% of centers that reported limited or no access to ocular prostheses. Induction chemotherapy can also be used as a strategy to facilitate enucleation in LMICs when parents are reluctant to proceed at diagnosis.^6^ Addressing these challenges in the East and Mediterranean regions may enhance the ability for curative management in patients who require enucleation.

Ophthalmology availability and training shortages are not limited to the East and Mediterranean. A global survey conducted by the International Council of Ophthalmology revealed an average of 3.7 ophthalmologists per million in low-income countries, as opposed to an average of 76.2 ophthalmologists per million in high-income countries.^21^ This may affect our findings as to why ophthalmic resources, specifically focal therapy and local delivery of chemotherapy, were viewed with less confidence across the region in comparison with oncologic resources. Education through regional fellowships, specifically ocular oncology, is required to build retinoblastoma-specific expertise that is often sparingly included in general ophthalmology residencies.^22-24^ Le et al^23^ evaluated Canadian ophthalmology residency programs and found that ocular oncology represented one of the least number of days spent in rotation (< 60 days, compared with > 124 days for pediatric ophthalmology), and only 45% of residents assisted in the required five retinoblastoma enucleation surgeries throughout their training. Our assessment identified only two separate ocular oncology fellowships, one in Jordan and one in Egypt. Utilization of these programs could allow for expansion of retinoblastoma care and collaboration across the region.

Telemedicine offers an additional avenue for education. Telemedicine has proved successful in improving the survival of children with pediatric cancer by pairing experienced centers with those with less experience.^24^ A joint venture between King Hussein Cancer Center in Amman, Jordan, and St Jude Children’s Research Hospital established a multidisciplinary team, invested in ophthalmic infrastructure, and engaged in telemedicine videoconferencing and exchange visits, reducing retinoblastoma-specific mortality in Jordan from 40% to 4% within a decade.^24,25^

Stratification of survey respondents was based on ophthalmic resources because all centers had basic pediatric oncology infrastructure. Thus, diagnostics including MRI and therapeutics such as systemic chemotherapy, key components of a pediatric cancer unit, did not differentiate across tiers.^26^ However, palliative care, which is more specialized, was increased at centers with more specialized resources (among tier 3 centers compared with tier 1 and 2 centers; Table 3). Palliative care has only recently been incorporated into the landscape of pediatric oncology and can be restricted as a result of financial constraints, opioid availability, and lack of recognition of palliative care as a necessary subspecialty in LMICs.^27^ It is desirable that palliative care would be available across all tiers given the increased likelihood of advanced disease at diagnosis in LMICs.^18-20^

Aims and guidelines for each tier can be developed that prioritize first the life of the child, followed by ocular salvage and then vision. Chantada et al^6^ published recommendations for graduated-intensity guidelines for the treatment of retinoblastoma in developing countries, which can contribute to improved patient outcomes.^28^ Because of the unavailability of an ophthalmologist to treat retinoblastoma, the primary objective of tier 1 centers should be prompt referral of identified leukocoria. Primary care providers and families must learn to recognize the leukocoria, or white pupil, the most common presenting symptom of retinoblastoma. An integrated regional network can lead to prompt referral of these patients to a multidisciplinary center for management. An important step in this process is educational programs. Example campaigns in Honduras linked education regarding leukocoria to vaccinations, reducing the incidence of extraocular disease at diagnosis by 38%.^29^ The six identified tier 1 centers were either general children’s hospital or general oncology hospitals. Because of the nature of the question, a respondent indicating “no ophthalmologist available to treat retinoblastoma,” does not exclude the possibility of having an in-house ophthalmologist. Tier 1 centers may have an ophthalmologist, especially at the general children’s hospitals. In such a case, the primary objective should be early diagnosis and referral. We should emphasize that early diagnosis in this setting pertains to intraocular disease without the manifestations of inflammation, buphthalmos, and proptosis.

Tier 2 centers represented the largest number of respondents (n = 12). Tier 2 centers can treat advanced disease by primary enucleation (with a pathologist to evaluate an enucleated eye to determine risk and need for adjuvant chemotherapy after surgery) or defer enucleation until chemotherapy has been given. Neoadjuvant chemotherapy may be required to reduce buphthalmos or allow time for the parents to accept surgical recommendations.^6,30^ Buphthalmos is present in two thirds of patients with retinoblastoma at presentation in LMICs,^31^ and enucleation holds a greater risk of globe rupture with disease spread.

Five respondents were identified as tier 3 centers possessing comprehensive oncology and ophthalmology resources as well as fully integrated multidisciplinary teams. Conservative eye-sparing therapy is a principal objective for tier 3 centers for patients amendable to chemoreduction with focal consolidation, especially when follow-up is likely and in the case of bilateral disease. These tier 3 centers can thus become a hub for patient care and retinoblastoma education. This was modeled by the Children’s Cancer Institute in Beirut, Lebanon, which increased referrals from other centers in Lebanon, Syria, and Iraq after establishment of a program in 2012.^15^ Regional hubs, as opposed to remote partners, are more accessible and apt to address cultural barriers, facilitating care of the child. These centers can also build educational capacity throughout the region by identifying themselves as centers of excellence, serving as leaders in the management of retinoblastoma in the region.

This assessment was subject to limitations. First, there was a low response rate of 32% from POEM member countries at the time of distribution. Although this sample size is good for the methodology, 68% of centers still failed to respond. Second, we must recognize the inherent bias that comes along with any survey instrument. This includes the bias of self-reporting answers and self-interpretation of questions. Investigating resource data in relation to patient outcomes is of useful inquiry for future studies.

This assessment revealed common themes related to the treatment of pediatric cancer, more specifically retinoblastoma, in LMICs, including decentralization of care, limited subspecialty expertise, and lack of available multidisciplinary care. Resource disparities warrant targeted intervention in the Eastern Mediterranean region to advance the management of retinoblastoma throughout the region. Such strategies include centralization of care, education through regional fellowships, recruitment of multidisciplinary teams, and establishment of regional centers of excellence.
